# Predictors of change in poor mental health and obesity (including overweight) comorbidity in mid-adolescence: A longitudinal analysis using the millennium cohort study

**DOI:** 10.1177/26335565261466283

**Published:** 2026-08-21

**Authors:** Afrin Khan, Alexis Karamanos, Amal R. Khanolkar

**Affiliations:** 1 Department of Population Health Sciences, School of Life Course & Population Sciences, 4616 King’s College London, London, UK

**Keywords:** adolescent health, obesity, depression, comorbidity, adverse childhood experiences, sexual minority

## Abstract

**Background:**

Depression and obesity are common chronic conditions in adolescence with major health consequences. While adverse childhood experiences (ACEs), socioeconomic disadvantage, and sexual minority (SM) identity are linked to higher mental health–obesity (MH-OB) comorbidity, evidence on predictors of change during adolescence is limited.

**Methods:**

Data came from 10,353 UK adolescents (49% male, 80.9% White, 9.1% SM) in the Millennium Cohort Study. MH-OB comorbidity was defined using overweight/obesity (International Obesity Task Force criteria) and depression/anxiety symptoms (Strengths and Difficulties Questionnaire emotional symptoms subscale) at ages 14 and 17. Change in comorbidity was categorised as none, persistent, resolved, or newly developed. Predictors included sex at birth, ethnicity, sexual identity, socioeconomic position, and cumulative ACEs. Multinomial logistic regression estimated adjusted relative risk ratios (aRRRs).

**Results:**

At age 14, 7.7% of adolescents were obese, 19.5% overweight, and 27.8% reported depression/anxiety symptoms. By age 17, 2.5% had persistent MH-OB comorbidity, 6.2% newly developed comorbidity, and 2.8% resolved; 88.0% had no comorbidity. Female sex, SM identity, lower household income, and increasing ACE exposure were associated with persistent and newly developed comorbidity. Associations were strongest among SM adolescents and females. Exposure to three or more ACEs increased risk of persistent comorbidity.

**Conclusion:**

Adolescents who experienced socioeconomic disadvantage, exposure to adversity, and with SM identity were more likely to show persistent or new-onset MH-OB comorbidity, with associations generally stronger in females than in males. These findings highlight the importance of early, integrated interventions that address both mental health and weight-related risks in structurally disadvantaged groups.

## Introduction

Obesity and poor mental health (like depression and anxiety) are significant contributors to the global burden of disease, especially during adolescence which is a crucial developmental phase characterized by significant physical, psychological, and social transformations. These conditions frequently originate in childhood, with a strong propensity to track into adulthood, and are associated with short- and long-term health outcomes, including chronic diseases (like type 2 diabetes and cardiovascular disorders), social disadvantage, and diminished economic opportunities.^1–3^ The prevalence of both conditions among adolescents has risen markedly in recent decades. In the UK, the 2021-22 National Child Measurement Programme reported that 10.1% of children in Reception (aged 4–5 years) and 23.4% in Year 6 (aged 10–11 years) were living with obesity, a sharp increase from pre-pandemic levels.^4^ Mental health has followed a similar trajectory; data from NHS Digital’s 2023 survey revealed that 20.3% of children aged 8–16 and 23.3% aged 17–19 were identified as having a probable mental disorder.^5^ Obesity and mental health problems are leading causes of disability among youth and major contributors to global morbidity, posing significant public health challenges.^6^

The co-occurrence of obesity and poor mental health (MH-OB comorbidity) is a pressing public health issue due to compounded negative effects. A recent global systematic review and meta-analysis estimated that 21% of children and adolescents are affected by overweight or obesity, with prevalence significantly higher among those exhibiting co-occurring depressive symptoms.^7^ Another review highlighted the prevalence of obesity among children and adolescents with depression, including its significant role as a prognostic factor influencing treatment outcomes and long-term health.^8,9^ The relationship between these conditions is bidirectional: obesity may trigger depression through stigma, lower self-esteem, and reduced physical activity, whereas depression may lead to obesity through negative lifestyle choices (like reduced physical activity, change in dietary patterns) and medication side-effects.^9–11^ Shared etiological factors including genetic vulnerabilities, adverse health behaviours, and socioeconomic adversity can further complicate the relationship and increase risk for MH-OB comorbidity.^12,13^ However, despite robust evidence from adult populations, longitudinal studies examining determinants of MH-OB comorbidity specifically during adolescence remain sparse.

Adverse childhood experiences (ACEs), encompassing abuse, neglect, parental psychological distress, and bullying are determinants of both short- and long-term ill-health, including obesity, poor mental health, chronic illness (including multimorbidity), and health-risk behaviours across the life course.^14–19^ The prevalence of ACEs is notably high (depending on the types of ACEs being examined and how they are measured), with approximately half of the global population reporting at least one ACE. ACEs are more common among socioeconomically disadvantaged, sexual minority and ethnic minority groups. Short-term consequences of ACEs in adolescence include obesity, depression, substance misuse, and reduced educational achievement.^20,21^ Bullying is one of the most reported ACEs and is strongly associated with increased adiposity in youth via stress-related coping behaviours like emotional eating and decreased physical activity, consistent with the diathesis-stress model.^22^

Ethnicity and sexual minority identity often intersect and cluster with other risk factors—such as socioeconomic disadvantage, ACEs, barriers in accessing healthcare, and the obesogenic environment—amplifying and perpetuating disparities in both mental health and obesity in childhood.^23^ In the UK, associations between ethnicity and mental health problems in childhood are complex and vary by ethnic group and type of mental health problem.^24^ For example, some studies show that most ethnic minority children have lower risk for internalising problems like depression and anxiety compared to White peers, while other studies show no differences in externalising and conduct problems between White and most ethnic minority children. These associations are further complicated by intersectional associations with patterns varying across groups and shaped by complex social, cultural, and biological influences. On the other hand, patterns of association with obesity in adolescence are more consistent, with adolescents from socioeconomically disadvantaged and ethnic minority backgrounds consistently having higher prevalence of obesity due to persistent disadvantage including living in deprived neighbourhoods, greater ACE exposure and limited access to protective resources.^25^ UK’s data shows stark socioeconomic disparities, with obesity prevalence nearly twice as high among adolescents from deprived backgrounds compared to their affluent peers (31.3% versus 13.5%).^26^ Similarly, adolescents identifying with sexual minority identities experience higher rates of stigma, victimisation, bullying, abuse, exacerbating their vulnerability to individually occurring and MH-OB comorbidity.^27^ Ethnic disparities in ACE exposure and associated health outcomes further emphasize the need for tailored public health strategies.^28^

Public health approaches have historically targeted obesity and mental disorders individually, achieving limited success in reducing their prevalence or disparities. Addressing these conditions as interrelated phenomena, particularly during adolescence, requires comprehensive approaches including identifying predictors of longitudinal transitions in comorbidity status. Thus, exploring how cumulative ACE exposure, socioeconomic disadvantage, sex at birth and sexual identity predict the persistence, resolution, or new onset of MH-OB comorbidity during adolescence is critical.

### Comparison with existing literature

Longitudinal studies using nationally representative data to examine predictors of change in MH-OB comorbidity during adolescence are limited. While robust evidence links ACEs with obesity or mental health separately, few studies have examined how ACEs, alongside other social determinants like socioeconomic disadvantage and sexual identity, predict changes in comorbidity over time. For instance, a recent longitudinal study using the Millennium Cohort Study (MCS) data demonstrated dose-response associations between ACE exposure and worsening mental health trajectories.^29^ In addition, a meta-analysis of over 133,000 adolescents identified bullying as a key predictor of depression, especially among overweight adolescents, perpetuating cycles of poor mental and physical health.^30^ Racism and discrimination represent additional stressors that compound these disparities, with evidence showing that reported racism is associated with poorer health and wellbeing among ethnic minority children and young people.^31^ Evidence linking ACE exposure to adolescent obesity is mixed. Some MCS studies found that parental mental illness and cumulative adversity predicted steeper BMI trajectories,^32^ while international comparisons (UK vs. Brazil) reported inconsistent outcomes, suggesting effects may emerge more clearly in adulthood.^33,34^

Although numerous studies have examined mental health and obesity comorbidity in both adults and children, few have explored the predictors of change in comorbidity status during adolescence, a critical gap this study seeks to address. A recent study using the MCS data demonstrated significant associations between specific ACEs—particularly maternal psychological distress and bullying—and MH-OB comorbidity separately at ages 14 and 17.^35^ Building on this finding, this study aims to explore longitudinal changes in comorbidity status in mid to late adolescence.

Moreover, while sociodemographic factors like socioeconomic disadvantage, ethnicity, and sexual identity clearly influence ACE exposure and health outcomes, their role in longitudinal changes in MH-OB comorbidity remains understudied. Research has identified sexual minority identity as being associated with increased risk for concurrent poor mental health and obesity, partly due to elevated exposure to ACEs.^35^ Similarly, ethnic variations in ACE exposure suggest complex intersections influencing MH-OB comorbidity currently not fully understood.^36^

Despite extensive evidence on associations between socioeconomic deprivation, ACEs and either obesity or mental health problems, very few studies have examined how these adversities jointly shape changes in MH–OB comorbidity in adolescence. Existing research has largely been cross-sectional or has not modelled dynamic changes over time, leaving a substantive gap in understanding why some adolescents have persistent comorbidity while others no longer have comorbidity. Using data from a large nationally representative cohort, this study provides a more nuanced characterisation of the social and demographic determinants associated with different comorbidity transitions and addresses methodological limitations in prior work. This study aimed to identify how socioeconomic position (SEP), ACE exposure, and sexual and ethnic identity predict changes in mental health–obesity comorbidity between ages 14 and 17 in a nationally representative adolescent cohort.

## Methods

### Study design and participants

This study utilized data from the Millennium Cohort Study (MCS), a nationally representative birth cohort, comprising children born across the UK between September 2000 and January 2002. 19,517 children were recruited initially with data collection conducted across seven sweeps: 9 months of age (MCS1) and at ages 3 (MCS2), 5 (MCS3), 7 (MCS4), 11 years (MCS5), 14 (MCS6) and 17 years (MCS7). Detailed information on the study, sampling and survey design can be found at: https://cls.ucl.ac.uk/cls-studies/millennium-cohort-study. Briefly, a nationally representative birth cohort from across the UK were recruited to the MCS and included children living in non-household situations and those not born in the UK but lived in the country at time of recruitment. The sampling strategy was to recruit 100% of eligible children living in geographically defined areas of residence (the boundaries of electoral wards as defined before the 2001 national census) during the eligible period. A stratified cluster sampling design was used to ensure adequate representation of families living in disadvantaged areas and from ethnic minority groups. Attrition across follow-up was predicted by single-parent families, parents with lower-income occupation and lower educational level, Black ethnicity, and male sex. The Centre for Longitudinal Studies has conducted extensive analyses of attrition across MCS sweeps, consistently demonstrating that despite cumulative attrition of approximately 47%, the retained sample remains broadly representative of the original birth cohort.^37^ This study includes N = 10,734 children who attended the age 14 and age 17 assessments, having data on mental health and BMI at both sweeps and data on any ACE from the ages 3, 5, 7 and 11 sweeps. Of the 10,734 adolescents with BMI and mental health data available at both sweeps, a total of 10,353 were eligible for analysis. The reduction reflects exclusion of individuals with complete outcome data but missing all predictor variables (sex, ethnicity, household income, parental education, or cumulative ACE score). Participants with at least one predictor variable available were retained.

### Measures

#### Obesity/overweight measurement

Overweight and obesity were determined using body mass index (BMI), calculated from standardized height and weight measurements collected by trained interviewers at ages 14 and 17. Classification of overweight and obesity was based on the International Obesity Task Force (IOTF) age- and sex-specific BMI cut-offs, which align child BMI percentiles with adult BMI thresholds for overweight (≥25kg/m^2^) and obesity (≥30 kg/m^2^).

#### Mental health measures

Poor mental health symptoms were assessed using three validated self-report instruments at the two-time-points. At age 14, the Short Mood and Feelings Questionnaire (SMFQ)—a 13-item scale developed for children and adolescents—was used to assess depressive symptoms over the previous two weeks.^38^ Respondents rated items such as *“I felt miserable or unhappy”* and *“I didn’t enjoy anything at all”* on a 3-point Likert scale (not true=0, sometimes true=1, true=2), yielding a total score from 0 to 26, with higher scores indicating potential depression. A threshold of ≥8 was used to indicate elevated depressive symptoms based on previous validation studies^.39^

At age 17, two separate scales were used. The Kessler Psychological Distress Scale (K6) is a 6-item measure of nonspecific psychological distress over the past 30 days.^40^ Respondents rated the frequency of symptoms like feeling nervous or worthless on a 5-point Likert scale (none of the time=0, little of the time=1, some of the time=2, most of the time=3, all of the time=4), with total scores ranging from 0 to 24 with a score of ≥13 considered to indicate probable serious poor mental health.^23^ The second scale was the Strengths and Difficulties Questionnaire (SDQ) Emotional symptoms subscale (SDQ-E), which includes five items on emotional difficulties experienced in the past six months.^41^ Each item was rated on a 3-point scale (not true=0, somewhat true=1, certainly true=2), producing a total score from 0 to 10 with scores ≥6 considered to indicate ‘high’ or ‘very high’ emotional difficulties, in line with UK guidance.^42^

Supplementary Table 1 provides a complete list of individual items, scoring categories, and response structures for each scale.

#### Poor mental health and overweight/obesity comorbidity status (MH-OB)

We created separate MH-OB comorbidity indicators at both ages 14 and 17. These indicators were binary and included adolescents without MH-OB comorbidity (coded as 0) and those with comorbidity (coded as 1), i.e., individuals having overweight/obesity *and* poor mental health (using the relevant cut-offs described above). Individuals who only had overweight/obesity or poor mental health were coded as having no comorbidity. At age 17, we created two comorbidity indicators using either mental health indicator (i.e., SDQ-E and K6).

#### MH-OB comorbidity changes between ages 14 and 17 (MH-OB)

The primary outcome for this study was a four-category measure of change in MH–OB comorbidity between ages 14 and 17. MH–OB comorbidity was defined as the simultaneous presence of overweight/obesity (based on age- and sex-adjusted BMI cut-offs) and poor mental health (using validated thresholds for the SMFQ at age 14 and SDQ-E *or* K6 at age 17). Using the binary comorbidity indicators at ages 14 and 17, we derived a categorical variable indicating four possible statuses in MH-OB comorbidity between ages 14-17: (1) No comorbidity, (2) Persistent comorbidity, i.e. having MH-OB comorbidity at both ages (3) Newly developed comorbidity, i.e., no comorbidity at age 14 but having comorbidity at age 17 and (4) Resolved comorbidity i.e., having comorbidity at age 14 but no longer at age 17. This four-level transition variable was the main dependent variable in all analysis.

We created two versions of the MH-OB comorbidity change variable, one using the SDQ-E and the other using the K6 mental indicator at age 17.

#### Adverse Childhood Experiences (ACEs)

ACEs were assessed prospectively from ages 3 to 11 and included maternal problem drinking, maternal drug use, parental psychological distress, intimate partner violence, physical punishment, and bullying (previously described in detail).^43^ All ACEs were reported by the primary caregiver, most commonly the mother, across multiple sweeps of the cohort. The only exception was bullying, which was self-reported by adolescents, providing subjective account of peer-related adversity. A cumulative ACE score was created by summing the six prospectively measured binary ACE indicators (not exposed=0 and exposed=1). Based on this cumulative ACE score, participants were categorised into four groups: 0 ACEs, 1 ACE, 2 ACEs, and ≥3 ACEs.^13^

#### Sociodemographic predictors

Sexual identity included heterosexual, bisexual, and gay/lesbian groups. Those who answered other or unknown sexual identity were excluded. Ethnicity was initially reported by a parent or guardian when the participant was aged three. If ethnicity data were missing at this time point, information from subsequent survey sweeps was used to supplement missing values. Participants were grouped into White (reference), mixed ethnicity, South Asian, Black and other ethnic groups. SEP was based on parental household income and highest parental education level. Household income was measured when the child was aged three and adjusted for household composition using the UK-equivalized income scale developed by the Organization for Economic Co-operation and Development. The resulting values were categorized into five income quintiles, ranging from quintile 1 (lowest income group) to quintile 5 (highest income group), allowing for relative comparisons across the cohort. Parental education was based on the highest academic or vocational qualification reported at baseline and categorized using the National Vocational Qualification (NVQ) framework, ranging from NVQ1 (lowest) to NVQ5 and above (highest levels including foundational degrees and diplomas, and university education). These two dimensions were modelled separately to capture both economic and educational aspects of early-life familial SEP. Household income and parental education were measured at age three, reflecting early-life familial socioeconomic position. Early childhood SEP (typically before ages five to six) is considered particularly influential on long-term health outcomes in life course epidemiology, and has been used in this way in previous MCS studies.^44^ Additionally, parental educational attainment is unlikely to change substantially over time for most individuals.

### Statistical analysis

Descriptive analyses were first conducted to summarise the distribution of all predictors and outcome categories, using weighted proportions and means based on the multiply imputed dataset (Table 1).

**Table 1.** Weighted descriptive characteristics of the analytical sample (N = 10,353 adolescents from the Millennium Cohort Study). Estimates are from multiply imputed data.

Predictors	Percentage (%)	95% CI	P value*
**Sex**			
Male	48.8	47.8–49.8	0.021
Female	51.1	50.1–52.1	
**Ethnicity**			
White	80.9	80.1–81.6	<0.001
mixed ethnicity	2.9	2.6–3.3	
South Asian	10.8	10.2–11.4	
Black	3.3	2.9–3.6	
other ethnic groups	1.8	1.6–2.1	
**Sexual identity**			
Heterosexual	79.6	78.8–80.4	<0.001
Mainly Heterosexual	11.1	10.5–11.7	
Bisexual	6.6	6.1–7.1	
Gay/Lesbian	2.5	2.2–2.9	
**Parental education** ^1^			
Overseas/Others	9.8	9.2–10.3	<0.001
NVQ1	4.4	4.0–4.8	
NVQ2	21.1	20.4–21.9	
NVQ3	15.2	14.5–15.9	
NVQ4	37.5	36.6–38.4	
NVQ5 (University/degree level)	11.5	11.1–12.3	
**Income in quintiles**			
1	20.3	19.5–21	<0.001
2	20.5	19.7–21.3	
3	19.9	19.1–20.7	
4	19.2	18.5–20	
5	19.8	19.1–20.6	
**BMI (kg/m** ^**2**^ **) at age 14**			
Not overweight (including underweight)	72.7	71.7–73.6	<0.001
Overweight	19.5	18.7–20.3	
Obese	7.7	7.2–8.3	
**BMI (kg/m** ^**2**^ **) at age 17**			
Not overweight (including underweight)	69.3	68.4–70.2	<0.001
Overweight	19.4	18.6–20.2	
Obese	11.1	10.5–11.8	
**Comorbidity status change (Kessler-6)**			
No Co-morbidity	88.3	87.6–89	<0.001
Persistent Co-morbidity	2.5	2.1–2.8	
Newly Developed Co-morbidity	2.8	2.4–3.2	
Resolved Co-morbidity	6.2	5.7–6.7	
**Comorbidity status change (SDQ-E)**			
No Co-morbidity	87	86.3–87.7	<0.001
Persistent Co-morbidity	3.1	2.7–3.5	
Newly Developed Co-morbidity	4.1	3.7–4.5	
Resolved Co-morbidity	5.6	5.1–6.1	
**Cumulative ACE Score** ^2^			
0	43.8	42.7–44.8	<0.001
1	29.3	28.2–30.4	
2	14.3	13.4–15.2	
≥3	12.5	11.4–13.6	

*Notes.*

^1^NVQ (National Vocational Qualification) levels: NVQ5 = university degree or higher; NVQ4 = higher education below degree; NVQ3 = A-level or equivalent; NVQ2 = GCSE level; NVQ1 = basic qualification; “Overseas/Other” = qualifications not mapped to the UK NVQ framework.

^2^Adverse childhood experiences (ACEs) include maternal problem drinking, maternal drug use, parental psychological distress, intimate partner violence, physical punishment, and bullying collected prospectively between ages 3 and 11.

Estimates are based on multiply imputed data using MICE (N = 10,353) and survey weights from the birth sweep. P-values indicate statistical differences in proportions using chi-square test; CI = Confidence Interval and 95% CI represent precision around weighted proportions.

Multinomial logistic regression models assessed associations between predictors of interest (cumulative ACE scores, sex, parental income and education, ethnicity, sexual identity) and comorbidity status. Multinomial logistic regression modelling was chosen as it generalises the logistic regression to multiclass problems as with the outcome of interest (MH-OB comorbidity) which has >2 categories. More specifically it enabled us to estimate the chances for persistent, resolved, and newly developed comorbidity compared to the no comorbidity group, across the different categories of the predictors of interest. The multinomial logistic regression model estimates the relative risk ratio (RRR), which is the ratio of two relative risks (derived from the exponentiated multinomial logit coefficient) and is interpreted for a unit change in the predictor variables. Further information on interpretation of RRR (which, in the context of multinomial logistic regression with more than two outcome categories, can be interpreted as conditional odds ratios as per the Stata Technical Bulletin 53) is provided in the relevant Stata technical bulletin.^45^

Initially, unadjusted (crude) models were run to separately examine associations between each predictor of interest and comorbidity status between ages 14 and 17 (i.e. persistent, newly developed, resolved vs. no comorbidity). We then ran a second multivariable model including all predictors of interest (i.e., mutually adjusted for sex, sexual identity, ethnicity, parental income quintile and education level, and cumulative ACE score) to estimate independent but adjusted associations. Both the crude and adjusted regression models were run with both versions of the MH-OB status variables. As a sensitivity analysis, we also ran all models restricted to complete case analysis (*N* = 8,145 for SDQ-E and *N* = 8,148 for Kessler-6.)

### Imputation methods for missing data

Due to attrition over follow-up, the extent of missing data varied across variables. Missing data on adverse childhood experiences ranged from 17.4% for bullying to 48.5% for parental drinking and 46.1% for exposure to violence, with 34.8% missing for maternal depression, 25.1% for maternal drug use, and 24.8% for physical punishment (smacking). In contrast, missing data for key sociodemographic variables was minimal, with 0.1% for household income and ethnicity, and 0.03% for parental education.

Missing data were addressed using Multiple Imputation by Chained Equations (MICE) under the Missing at Random (MAR) assumption.^46^ The imputation model included all analytic variables—ACEs, sociodemographic covariates, and outcome variables as well as a set of auxiliary variables not used in the main analysis but predictive of missingness (e.g., maternal age at birth, household structure, and indicators of racism or discrimination) to help improve the precision of imputed data. Interaction terms between individual ACEs, sex, ethnicity, parental income, and sexual identity were also included to ensure compatibility between the imputation and analysis models. Missingness was substantially higher for some auxiliary variables not used as primary exposures or outcomes; for example, perceived racism reported at age 5 had over 80% missing data. Given the very high missingness for certain auxiliary variables (e.g., perceived racism at age 5), we generated eighty imputed datasets to improve the stability of estimates; imputed estimates were pooled using Rubin’s rules to obtain valid standard errors and confidence intervals.^47,48^

To account for the stratified cluster design of the MCS and attrition over time, all models were weighted using non-response weights from the birth sweep. The Stata *‘svy’* command was used for survey data analysis. Relative Risk Ratios (RRRs) from the multinomial logistic regression models were plotted for visualisation, and analyses were conducted in Stata V17 (StataCorp LP, College Station, Texas).

## Results

Table 1 presents descriptive statistics for key sociodemographic, ACEs, and MH-OB comorbidity variables at baseline (age 14) and follow-up (age 17). The prevalence of overweight remained stable between ages 14 and 17. However, the prevalence of obesity increased from 7.7% at age 14 to 11.1% at age 17. MH-OB comorbidity (using the SDQ-E indicator) showed that 87.0% had no comorbidity across ages 14 and 17, while 3.1% had persistent comorbidity, 4.1% newly developed, and 5.6% resolved comorbidity. Comparable patterns were observed using the K6 indicator of mental health. A majority (43.8%) reported no ACEs, while 29.3% had experienced one, 14.3% had two, and 12.5% had three or more ACEs (Table 2).

**Table 2.** Associations between early–life predictors and change in poor mental health–obesity comorbidity between ages 14 and 17 in 10,353 adolescents from the UK Millennium Cohort Study. Estimates are from multinomial logistic regression models.

Predictor	Comorbidity status change	Unadjusted RRR (95% CI)	Adjusted RRR (95% CI)^1^
Sex at birth	Male	Reference	
	**No comorbidity**	Base outcome	
	**Persistent comorbidity**		
	Female	**4.70 (3.46–6.39)**	**4.15 (3.04–5.67)**
	**Newly developed comorbidity**		
	Female	**2.51 (1.99–3.18)**	**2.28 (1.79–2.89)**
	**Resolved comorbidity**		
	Female	**1.78 (1.47–2.16)**	**1.77 (1.45–2.16)**
Ethnicity	White	Reference	
	**No comorbidity**	Base outcome	
	**Persistent comorbidity**		
	mixed ethnicity	0.93 (0.45–1.92)	0.79 (0.37–1.67)
	South Asian	**0.48 (0.28–0.83)**	**0.48 (0.27–0.85)**
	Black	0.93 (0.49–1.78)	0.94 (0.47–1.86)
	other ethnic groups	0.29 (0.07–1.19)	0.25 (0.06–1.04)
	**Newly developed comorbidity**		
	mixed ethnicity	1.28 (0.74–2.24)	1.08 (0.61–1.91)
	South Asian	**0.63 (0.42–0.94)**	**0.55 (0.36–0.84)**
	Black	0.49 (0.21–1.12)	0.43 (0.18–1.00)
	other ethnic groups	0.61 (0.25–1.49)	0.50 (0.20–1.27)
	**Resolved comorbidity**		
	mixed ethnicity	1.32 (0.82–2.12)	1.17 (0.72–1.90)
	South Asian	0.78 (0.56–1.07)	**0.66 (0.46–0.94)**
	Black	1.22 (0.74–2.03)	1.05 (0.62–1.78)
	other ethnic groups	0.75 (0.35–1.64)	0.61 (0.28–1.35)
Sexual identity	Heterosexual	Reference	
	**No comorbidity**	Base outcome	
	**Persistent comorbidity**		
	Mainly heterosexual	**3.09 (2.24–4.26)**	**2.84 (2.04–3.95)**
	Bisexual	**5.31 (3.80–7.42)**	**4.02 (2.85–5.67)**
	Gay/Lesbian	**6.69 (4.14–10.80)**	**5.93 (3.62–9.72)**
	**Newly developed comorbidity**		
	Mainly heterosexual	**2.20 (1.65–2.94)**	**2.06 (1.53–2.77)**
	Bisexual	**3.30 (2.39–4.55)**	**2.62 (1.88–3.65)**
	Gay/Lesbian	**2.77 (1.62–4.75)**	**2.49 (1.45–4.30)**
	**Resolved comorbidity**		
	Mainly heterosexual	**1.61 (1.24–2.10)**	**1.57 (1.20–2.06)**
	Bisexual	1.43 (1.00–2.05)	1.26 (0.88–1.82)
	Gay/Lesbian	**1.92 (1.16–3.18)**	**1.81 (1.09–3.00)**
Income in quintiles	Q4 (highest)	Reference	
	**No comorbidity**	Base outcome	
	**Persistent comorbidity**		
	Q0 (lowest)	**1.78 (1.17–2.73)**	1.55 (0.92–2.60)
	Q1	**2.06 (1.37–3.10)**	**1.81 (1.13–2.89)**
	Q2	**1.69 (1.10–2.59)**	1.56 (0.98–2.49)
	Q3	**1.67 (1.09–2.55)**	**1.58 (1.02–2.45)**
	**Newly developed comorbidity**		
	Q0 (lowest)	**1.62 (1.14–2.30)**	**1.91 (1.25–2.92)**
	Q1	**1.71 (1.22–2.39)**	**2.00 (1.36–2.94)**
	Q2	1.41 (0.98–2.01)	**1.59 (1.08–2.33)**
	Q3	1.09 (0.75–1.59)	1.15 (0.78–1.70)
	**Resolved comorbidity**		
	Q0	**1.64 (1.21–2.22)**	1.36 (0.94–1.97)
	Q1	**1.49 (1.10–2.03)**	1.31 (0.92–1.87)
	Q2	**1.62 (1.20–2.19)**	**1.51 (1.09–2.09)**
	Q3	1.29 (0.94–1.77)	1.23 (0.89–1.71)
Parental Education (NVQ Levels)	NVQ5 (Degree Level)	Reference	
	**No comorbidity**	Base outcome	
	**Persistent comorbidity**		
	Overseas/other	1.42 (0.78–2.57)	1.37 (0.69–2.71)
	NVQ 1	1.88 (0.95–3.69)	1.64 (0.78–3.44)
	NVQ 2	**1.85 (1.15–3.00)**	1.57 (0.92–2.69)
	NVQ 3	**2.00 (1.22–3.27)**	**1.81 (1.06–3.11)**
	NVQ 4	1.32 (0.83–2.10)	1.27 (0.78–2.05)
	**Newly developed comorbidity**		
	Overseas/other	1.53 (0.96–2.43)	1.28 (0.74–2.21)
	NVQ 1	1.55 (0.88–2.74)	1.15 (0.62–2.13)
	NVQ 2	1.38 (0.92–2.06)	1.04 (0.66–1.62)
	NVQ 3	1.09 (0.70–1.69)	0.89 (0.55–1.44)
	NVQ 4	1.31 (0.91–1.89)	1.22 (0.83–1.79)
	**Resolved comorbidity**		
	Overseas/other	**1.60 (1.06–2.41)**	1.35 (0.83–2.18)
	NVQ 1	1.41 (0.82–2.45)	1.17 (0.65–2.10)
	NVQ 2	**1.71 (1.21–2.42)**	1.43 (0.98–2.10)
	NVQ 3	1.34 (0.92–1.95)	1.16 (0.77–1.73)
	NVQ 4	1.26 (0.91–1.76)	1.18 (0.84–1.66)
Cumulative ACE score	**ACE score 0**	Reference	
	**No comorbidity**	Base outcome	
	**Persistent comorbidity**		
	1	1.08 (0.78–1.51)	1.18 (0.83–1.68)
	2	1.33 (0.91–1.95)	**1.53 (1.01–2.33)**
	≥3	1.32 (0.82–2.11)	1.65 (0.95–2.87)
	**Newly developed comorbidity**		
	1	1.07 (0.80–1.44)	1.12 (0.83–1.52)
	2	1.07 (0.75–1.54)	1.12 (0.76–1.64)
	≥3	1.09 (0.72–1.65)	1.16 (0.72–1.89)
	**Resolved comorbidity**		
	1	1.18 (0.92–1.52)	1.21 (0.94–1.56)
	2	**1.42 (1.06–1.91)**	**1.47 (1.07–2.00)**
	≥3	**1.43 (1.02–1.99)**	**1.50 (1.01–2.22)**

*Note.*

^1^Model mutually adjusted for sex at birth, ethnicity, sexual identity, parental income, parental highest educational level and adverse childhood experiences (ACE) score, with ‘no comorbidity’ as the reference category. Text in bold indicates 95% CIs which do not include 1.

Mental health is based on the Strengths and Difficulties Questionnaire (SDQ) – Emotional symptoms subscale. All models are run using imputed data and are survey weighted. RRR = Relative Risk Ratio; CI = Confidence Interval. Parental educational levels: NVQ5 = university degree or higher; NVQ4 = higher education below degree; NVQ3 = A–level or equivalent; NVQ2 = GCSE level; NVQ1 = basic qualification; “Overseas/Other” = qualifications not mapped to the UK NVQ framework.

We observed significant changes in the prevalence of and change in MH-OB comorbidity between ages 14 and 17. First, considering comorbidity at each sweep separately, 7.1% of adolescents had MH-OB comorbidity at age 14 using the SDQ-E indicator (6.4% using the K6 indicator of mental health). By age 17, these estimates increased slightly to 8.7% (SDQ-E) and 7.3% (K6). The majority (88.3%) of adolescents had no comorbidity at either age, but 2.5% exhibited persistent comorbidity, 6.2% developed comorbidity between ages 14 and 17 (i.e., new onset), and 2.8% experienced resolution (no longer having comorbid MH-OB at age 17). The MH-OB comorbidity change indicator using SDQ-E yielded slightly higher prevalence estimates for both persistent (3.1%) and newly developed comorbidity (4.1%), compared to that using the K6.

In mutually adjusted models, sexual identity was the most significant and consistent predictor of persistent comorbidity. Compared to heterosexual adolescents, all sexual minority groups were substantially more likely to experience persistent comorbidity compared to heterosexual peers (gay/lesbian: aRRR 5.93, 95% CI: 3.62–9.72; bisexual: aRRR 4.02, 95% CI: 2.85–5.67; and mainly heterosexual: aRRR 2.84, 95% CI: 2.04–3.95). These patterns with sexual minority identity were also observed for new onset comorbidity; bisexual adolescents: aRRR of 2.62, 1.88–3.65, and gay/lesbian adolescents: aRRR 2.49, 1.45–4.30.

Compared to male, female adolescents were significantly more likely to experience adverse comorbidity transitions, for example, increased risk for persistent comorbidity (aRRR 4.15, 3.04–5.67), and developing new comorbidity (aRRR 2.28, 1.79–2.89), but were also more likely to experience resolved comorbidity (aRRR 1.77, 1.45–2.16).

Household income gradients were evident, with adolescents from the lowest income quintile experiencing a nearly two-fold increased risk in developing comorbidity (aRRR 1.91, 1.25–2.92) and a moderately increased risk of persistent comorbidity (aRRR 1.55, 0.92–2.60), compared to those in the highest income quintile. Middle-income groups (quintiles 1–3) also experienced increased risk, with aRRRs ranging between 1.56 and 2.00 for developing MH-OB comorbidity.

Cumulative exposure to ACEs showed a dose–response pattern. Adolescents with two ACEs had significantly elevated risk of persistent comorbidity (aRRR 1.53, 1.01-2.33) but were also likely to experience resolved comorbidity (aRRR 1.47, 1.07-2.00). Adolescents with ≥3 ACEs also had increased risk for persistent comorbidity, but findings were not statistically significant (aRRR 1.65, 0.95-2.87). However, the association with resolved comorbidity remained significant (aRRR 1.50, 95% CI: 1.01-2.22).

Ethnic differences in comorbidity change were less pronounced. For example, South Asian adolescents had significantly lower risk of persistent (aRRR 0.48; 0.27–0.85) and new onset comorbidity (aRRR 0.55; 0.36–0.84) compared to White adolescents. Parental education was inconsistently associated with comorbidity transitions. Lower qualifications (eg: NVQ3 vs NVQ5) were significantly associated with persistent comorbidity (aRRR 1.81; 1.06-3.11), but associations for newly developed and resolved comorbidity were attenuated and no longer statistically significant in mutually adjusted models.

In the unadjusted (crude) models, most predictors, including sexual identity, sex at birth, household income, parental education, ACEs, and ethnicity showed statistically significant associations with comorbidity transitions like those in mutually adjusted models.

The SDQ-based models were described in detail above due to their slightly broader symptom scope (emotional symptoms over the previous six months) and higher sensitivity to chronic internalising problems in adolescence.^41^ However, models with MH-OB comorbidity using the K6 scale produced largely similar patterns of associations with all predictors (see Supplementary Table 2), supporting the robustness of findings observed with the SDQ. The direction and magnitude of all major associations were consistent with those from the SDQ. For example, gay/lesbian adolescents had an aRRR of 5.27 for persistent comorbidity in K6 models. Female sex also remained a strong predictor of persistent comorbidity (K6 aRRR 3.93 vs SDQ aRRR 4.15), and cumulative ACEs and low income were significantly associated with both persistent and newly developed comorbidity.

## Discussion

### Summary of main findings

This study provides one of the first longitudinal analyses of change in poor mental health–overweight/obesity (MH-OB) comorbidity from mid to late adolescence, using a two-time-point transition framework and nationally representative UK data. We observed clear patterns of persisting and new onset of MH-OB comorbidity between ages 14 and 17, particularly among sexual minority and female adolescents, those from lower-income households, and individuals exposed to multiple ACEs building directly on previous cross-sectional findings.^43^

In this longitudinal study of 10,353 adolescents from the Millennium Cohort Study, we examined transitions in mental health–obesity (MH-OB) comorbidity between ages 14 and 17, extending prior research that assessed comorbidity at single time points. Using a four-category outcome (persistent, newly developed, resolved, and no comorbidity), our study revealed that female sex, sexual minority identity (particularly in gay/lesbian and bisexual adolescents), lower household income, and cumulative ACE exposure (≥3 ACEs) were independently associated with greater risk of persistent and newly developed comorbidity. These associations were consistent with both the SDQ-E and Kessler-6 mental health indicators. Notably, associations with ACEs showed a significant dose–response relationship (gradient) in adjusted models, underscoring their substantial influence on adolescent health. The dynamic categorisation of change in comorbidity status between ages 14 and 17 provides a more granular understanding of how early-life adversity and social inequalities interact to influence health in adolescence.

The interpretation of ‘resolved comorbidity’ warrants careful consideration. Resolution may occur through improvement in mental health, reduction in weight status, or simultaneous recovery in both domains. Prior research suggests that psychological distress can resolve more rapidly than obesity, whereas weight reduction in adolescence often requires sustained behavioural or environmental change^.49^ Likewise, improved mental health may increase motivation and capability for healthier behaviours, helping in reducing BMI. Without direct measurement of mediators, we cannot determine the pathways that lead to reduction in MH-OB comorbidity, but recognising these distinct pathways is essential for understanding heterogeneous adolescent health trajectories.^50^

### Strengths and limitations

A major strength of this study is the longitudinal design, which allows for an in-depth analysis of predictors of comorbidity in mid to late adolescence. The classification of persistent, newly developed, resolved, and no comorbidity provides greater insight into the progression and resolution of comorbidity over time. The large sample size of 10,353 participants, combined with rich social and economic health data, enhances the generalizability of findings to the broader UK adolescent population.

Another strength is the use of prospectively collected ACE data, which minimizes the risk of recall bias and reverse causation. The study benefits from widely used and validated measures of depression and anxiety in adolescents and objectively measured BMI, reducing self-report bias commonly found in studies relying on self-reported BMI. Furthermore, by incorporating two different mental health scales (SDQ-E and K6), we ensured robustness and comparability in the assessment of MH-OB comorbidity and reduced the possibility of chance findings.

We considered cumulative ACE scores, offering distinct advantages and limitations. Using a total ACE score enables comparisons with studies due to its widespread use but this approach assumes that all ACEs have equal weight in influencing outcomes, which is unlikely.^51^ A key limitation was the inability to include important adversities such as sexual abuse or identity-based discrimination related to ethnicity or sexual identity as this data was not collected in the MCS. Previous studies have shown that sexual minority individuals, including in the MCS, report higher levels of bullying compared to heterosexual peers.^21^ Additionally, as approximately 95% of the respondents were mothers, we were unable to assess the specific effects of paternal mental health, risky behaviours, or parental adversities occurring outside the immediate family, as these were not captured in the MCS. Some ACEs like parental alcohol misuse may be underestimated due to reporting bias leading to misclassification. Similarly, the tools used to assess parental discord and harsh parenting based on the original Golombok Rust Inventory of Marital State scale only included a limited number of relevant items.

Like most longitudinal studies, MCS is subject to attrition, with 10,757 participants remaining at age 17 of the original 19,517 children recruited at birth. However, studies indicate that the age 17 sample remains broadly representative of the original cohort.^37^ While a formal comparison of baseline characteristics between retained and lost participants was not conducted within this study, the Centre for Longitudinal Studies has extensively documented attrition patterns across MCS sweeps, consistently showing the sample remains broadly representative despite cumulative attrition of approximately 47%. Missing data (item response) was addressed using validated multiple imputation (MI). While imputation assumes data is Missing at Random (MAR)—a condition that cannot be empirically verified—the plausibility of this assumption was strengthened by incorporating auxiliary variables, including maternal age at birth and vicarious racism at age 5, in the imputation model. These auxiliary variables improve the precision of missing data predictions and reduce non-random variation in values. Additionally, repeated measures of ACEs, BMI, and mental health—collected prospectively across multiple sweeps were included as predictors in the imputation model, strengthening the plausibility of the MAR assumption and improving the accuracy of imputed values. Additionally, although weighting and imputation were applied, attrition is known to disproportionately affect individuals with behavioral difficulties, mental health problems, or unstable home environments. This selective loss may bias estimates and should be considered when interpreting findings.

A further limitation relates to the generalisation of findings from this study to the current generation of adolescents in the UK. The MCS participants belong to the early ‘Millennial’ generation and their experiences including heightened exposure to social media, evolving norms around identity expression, and post-2008 economic uncertainty may differ from those of earlier or later cohorts. Caution is therefore required when generalising these findings to current adolescents.

Despite a large sample size, the number of adolescents with MH-OB comorbidity was small (8.4% at age 14 and 6.9% at age 17). This precluded analysis of estimating change in comorbidity status in intersecting identity groups (for example, running models with interactions with sex, sexual identity, ethnicity, and SEP). This needs to be explored in future studies with sufficiently large samples. A further limitation is that socioeconomic position was operationalized using household income and parental education measured at age three. While early-life SEP is well established as a key determinant of long-term health, this approach does not capture dynamic changes in socioeconomic circumstances across childhood and adolescence. Future studies should consider time-varying SEP measures to better understand how changes in socioeconomic conditions shape MH-OB comorbidity trajectories.^52^

Findings from the complete case analysis were broadly consistent with those from the multiply imputed models, with the direction and approximate size of the relative risk ratios remaining similar across key predictors. While a few estimates lost statistical significance in complete case (CC) analysis due to reduced power, the same key predictors-sexual minority identity, female sex, lower household income, and cumulative ACE exposure remained associated with increased risks of persistent and new-onset comorbidity. This consistency reinforces the robustness of the observed associations despite missing data. However, multiple imputation (MI) strengthened the association between high cumulative ACEs (≥3) and persistent comorbidity, revealing statistically significant associations not observed in CC. While results for income quintiles and ethnicity varied slightly, the direction of associations remained stable. MI produced slightly wider confidence intervals and higher standard errors, as expected, but overall findings were consistent, indicating that MI reliably accounted for missing data without altering the core conclusions.

Despite these methodological strengths, residual confounding remains a limitation, as unmeasured factors such as genetic predisposition, early-life nutrition, and disparities in healthcare access, neighbourhood factors can also influence MH-OB comorbidity development in adolescence. Additionally, self-reported mental health data may introduce measurement bias, as individuals may under- or over-report symptoms based on personal perceptions or social desirability. Nonetheless, the use of validated mental health measures, objectively measured BMI, and robust statistical methods enhances confidence in the reliability of our findings. Future studies should also examine predictors and change in MH-OB using clinically diagnosed mental health problems. A further limitation relates to the use of different mental health instruments across time points. At age 14, the SMFQ assessed depressive symptoms over a two-week recall period, whereas at age 17 the SDQ-E and K6 captured emotional symptoms over six months and 30 days respectively, with differing symptom domains and thresholds. This measurement non-equivalence is an inherent constraint of the MCS design and may contribute to observed changes in comorbidity status between sweeps. The use of two mental health indicators at age 17, which produced consistent findings, partially mitigates this concern.

Another limitation is that both sexual identity and MH-OB were assessed at the same timepoint of age 17 (and consequentially overlaps with the MH-OB change outcome). This impacts the temporality between sexual identity as a predictor of MH-OB change between ages 14 and 17. However, sexual identity evolves over many years, with studies showing that majority of individuals are aware of their sexual identity by early adolescence.^53^ It is reasonable to assume that sexual identity measured at age 17 reflects an identity conceived over time and most likely in early adolescence.

### Implications for public health and clinical practice

This study highlights a need to support adolescents who are living with MH-OB comorbidity. These young people often face intersecting vulnerabilities including socioeconomic hardship, early life adversity, and marginalisation related to sex and/or sexual identity which can compound over time adversely affecting wellbeing across the life course. Targeted and inclusive interventions are needed to address these risks in the general adolescent population and those with intersecting identities. For those working in healthcare and public health, the message is clear: prevention and support strategies must be more integrated and inclusive. This means not only improving access to mental health and weight management services but also embedding trauma-informed and culturally sensitive approaches within everyday practice. A one-size-fits-all model will not work; instead, responses should reflect the complexity of young people’s lived experiences.

The increased risks for MH-OB comorbidity observed among sexual minority adolescents align with the Minority Stress Theory, which posits that chronic exposure to identity-related stigma, discrimination, and stress negatively impacts both psychological and physical health.^54^ These stressors may contribute simultaneously to depressive symptoms and unhealthy coping behaviours including unhealthy eating habits, reduced physical activity, and health-risk behaviours which can lead to greater risk for MH–OB comorbidity. Recent work has shown that female sexual minority youth are particularly vulnerable to obesity through minority stress pathways, consistent with the patterns identified in this study.^55^

The present findings support the implementation of more targeted, actionable policy interventions within adolescent health services. First, routine social needs screening (covering food insecurity, family conflict, housing instability, and bullying experiences) could help identify adolescents at greatest risk of adverse MH–OB trajectories. Second, embedding ‘youth navigator’ roles within community and school-based health systems may improve linkage to mental health support, weight management services, and trauma-informed care, especially for adolescents facing intersecting disadvantages. Third, integrating screening for minority stress, peer victimisation, and ACE-related distress into routine adolescent assessments could facilitate earlier identification of comorbidity risk. These solutions reflect practical applications of the study’s findings and address the layered risk pathways underlying comorbidity. Recent multi-trajectory modelling using the MCS showed how structural disadvantage and family-level adversity co-evolve across childhood and adolescence to shape mental health outcomes. Distinct profiles of persistent poverty, material hardship, and cumulative adversity were shown to predict worsening mental health during adolescence, underscoring the interdependence of socioeconomic and psychosocial risk processes.^56^ This aligns with our finding that adolescents from lower-income households and those exposed to multiple ACEs experience higher risk for persistent or new-onset MH-OB comorbidity.

While this study sheds light on several predictors, there is still much to study. Future research should explore additional forms of adversity that were not captured here, such as racial discrimination, sexual abuse, and identity-based bullying, which may further shape young people’s health outcomes. It is also important to move beyond identifying risk and begin to understand why some adolescents recover while others continue to live with MH-OB comorbidity into late adolescence.

Changing dietary environments may contribute to higher risk for MH–OB comorbidity, particularly among socioeconomically disadvantaged adolescents. The increased availability of inexpensive, calorie-dense fast-food options often concentrated in low-income neighbourhoods has reshaped dietary patterns and amplified obesity risk. These environments intersect with psychological stress, emotional coping behaviours, and exposure to adversity, creating conditions that reinforce both poor mental health and unhealthy weight gain. Understanding comorbidity therefore requires attention not only to individual risk factors but also to the wider food systems shaping young people’s choices.^57,58^

In summary, this study found that sexual minority identity, socioeconomic disadvantage, and exposure to adversity were significantly associated with both persistent and developing MH-OB comorbidity in mid-to late adolescence, with associations generally stronger in female than in male adolescents. These findings highlight the importance of early, integrated interventions that address both mental health and weight-related risks in structurally disadvantaged groups.

## Supplemental material

Supplemental material - Predictors of change in poor mental health and obesity (including overweight) comorbidity in mid-adolescence: A longitudinal analysis using the millennium cohort study Supplemental material for Predictors of change in poor mental health and obesity (including overweight) comorbidity in mid-adolescence: A longitudinal analysis using the millennium cohort study by Afrin Khan, Alexis Karamanos, and Amal R. Khanolkar in Journal of Multimorbidity and Comorbidity.

## Data Availability

Data for this study is from the age 17 sweep of the Millennium Cohort Study. Data can be accessed from the UK Data Service website: https://ukdataservice.ac.uk/ (after registration and agreeing to licence terms and conditions).
