# Supplementary material for: Predictors of change in poor mental health and obesity (including overweight) comorbidity in mid-adolescence: A longitudinal analysis using the millennium cohort study
Source: J Multimorb Comorb. 2026 Aug 21;16:26335565261466283. doi: 10.1177/26335565261466283 (PMC13498805; doi:10.1177/26335565261466283)
Supplement: Supplementary file 1 [file sj-pdf-1-cob-10.1177_26335565261466283.pdf]

Supplementary Table 1. The mental health scales used in this study.

Scale	Item Number	Item Text	Response Scale
Short Mood and Feelings Questionnaire (SMFQ) – Age 14	1	I felt miserable or unhappy	0=Not true, 1=Sometimes true, 2=True
	2	I did not enjoy anything at all	0=Not true, 1=Sometimes true, 2=True
	3	I felt so tired I just sat around and did nothing	0=Not true, 1=Sometimes true, 2=True
	4	I was very restless	0=Not true, 1=Sometimes true, 2=True
	5	I was no good anymore	0=Not true, 1=Sometimes true, 2=True
	6	I cried a lot	0=Not true, 1=Sometimes true, 2=True
	7	I found it hard to think properly or concentrate	0=Not true, 1=Sometimes true, 2=True
	8	I hated myself	0=Not true, 1=Sometimes true, 2=True
	9	I was a bad person	0=Not true, 1=Sometimes true, 2=True
	10	I felt lonely	0=Not true, 1=Sometimes true, 2=True
	11	I thought nobody really loved me	0=Not true, 1=Sometimes true, 2=True
	12	I thought I could never be as good as other kids	0=Not true, 1=Sometimes true, 2=True
	13	I did everything wrong	0=Not true, 1=Sometimes true, 2=True
The Kessler Psychological Distress Scale (K6) – Age 17	1	During the past 30 days, how often did you feel nervous?	0=None of the time, 1=A little, 2=Some, 3=Most, 4=All the time
	2	During the past 30 days, how often did you feel hopeless?	0=None of the time, 1=A little, 2=Some, 3=Most, 4=All the time
	3	During the past 30 days, how often did you feel restless or fidgety?	0=None of the time, 1=A little, 2=Some, 3=Most, 4=All the time
	4	During the past 30 days, how often did you feel so depressed that nothing could cheer you up?	0=None of the time, 1=A little, 2=Some, 3=Most, 4=All the time
	5	During the past 30 days, how often did you feel that everything was an effort?	0=None of the time, 1=A little, 2=Some, 3=Most, 4=All the time
	6	During the past 30 days, how often did you feel worthless?	0=None of the time, 1=A little, 2=Some, 3=Most, 4=All the time
Strengths and	1	I get a lot of headaches,	0=Not true,

Difficulties Questionnaire (SDQ) Emotional symptoms subscale (SDQ-E) – Age 17		stomach-aches, or sickness	1=Somewhat true, 2=Certainly true
SDQ-ES	2	I worry a lot	0=Not true, 1=Somewhat true, 2=Certainly true
SDQ-ES	3	I am often unhappy, downhearted, or tearful	0=Not true, 1=Somewhat true, 2=Certainly true
SDQ-ES	4	I am nervous in new situations. I easily lose confidence	0=Not true, 1=Somewhat true, 2=Certainly true
SDQ-ES	5	I have many fears; I am easily scared	0=Not true, 1=Somewhat true, 2=Certainly true

Supplementary Table 2. Associations between early-life socioeconomic factors and changes in poor mental health–obesity comorbidity between ages 14 and 17, using the Kessler 6-item Psychological Distress Scale (K6), in 10,353 adolescents from the UK Millennium Cohort Study.

Predictor	Comorbidity Status Change	Unadjusted RRR (95% CI)	Adjusted RRR (95% CI)
**Sex at birth**	Male	Reference	
	**No comorbidity**	Base outcome	
	**Persistent comorbidity**		
	Female	4.53 (3.19–6.43)	3.93 (2.75–5.63)
	**Newly developed comorbidity**		
	Female	2.02 (1.54–2.64)	1.76 (1.33–2.32)
	**Resolved comorbidity**		
	Female	1.9 (1.58–2.29)	1.87 (1.55–2.27)
**Ethnicity**	White	Reference	
	**No comorbidity**	Base outcome	
	**Persistent comorbidity**		
	mixed ethnicity	1.26 (0.63–2.54)	1.09 (0.53–2.26)
	South Asian	0.63 (0.37–1.08)	0.64 (0.36–1.15)
	Black	1.15 (0.58–2.28)	1.19 (0.57–2.46)
	other ethnic groups	0.42 (0.10–1.68)	0.35 (0.08–1.49)
	**Newly developed comorbidity**		
	mixed ethnicity	1.43 (0.76–2.70)	1.12 (0.58–2.15)
	South Asian	0.84 (0.55–1.28)	0.69 (0.43–1.08)
	Black	0.91 (0.42–1.96)	0.76 (0.34–1.71)
	other ethnic groups	0.71 (0.25–1.99)	0.53 (0.18–1.52)
	**Resolved comorbidity**		
	mixed ethnicity	1.14 (0.70–1.85)	1.00 (0.61–1.65)
	South Asian	0.69 (0.51–0.95)	0.59 (0.42–0.84)
	Black	1.14 (0.70–1.85)	0.99 (0.60–1.65)
	other ethnic groups	0.66 (0.31–1.42)	0.54 (0.25–1.18)
**Sexual identity**	Heterosexual	Reference	
	**No comorbidity**	Base outcome	
	**Persistent comorbidity**		
	Mainly heterosexual	2.84 (1.92–4.20)	2.64 (1.77–3.94)
	Bisexual	6.22 (4.32–8.95)	4.87 (3.34–7.10)
	Gay/Lesbian	5.85 (3.40–10.04)	5.27 (3.03–9.16)
	**Newly developed comorbidity**		
	Mainly heterosexual	2.64 (1.88–3.71)	2.69 (1.90–3.81)
	Bisexual	4.43 (3.12–6.30)	3.90 (2.71–5.63)
	Gay/Lesbian	2.30 (1.09–4.86)	2.14 (1.00–4.57)
	**Resolved comorbidity**		
	Mainly heterosexual	1.77 (1.39–2.27)	1.72 (1.34–2.21)
	Bisexual	1.44 (1.03–2.02)	1.25 (0.89–1.76)
	Gay/Lesbian	2.37 (1.51–3.70)	2.19 (1.40–3.45)
**Income in quintiles**	Q4 (highest)	Reference	
	**No comorbidity**	Base outcome	
	**Persistent comorbidity**		
	Q0 (lowest)	2.31 (1.41–3.79)	1.84 (1.02–3.33)
	Q1	2.27 (1.39–3.72)	1.87 (1.07–3.27)
	Q2	2.30 (1.40–3.76)	2.08 (1.22–3.54)
	Q3	2.00 (1.21–3.30)	1.92 (1.14–3.22)
	**Newly developed comorbidity**		
	Q0 (lowest)	2.31 (1.51–3.52)	2.24 (1.34–3.73)
	Q1	2.04 (1.34–3.13)	2.07 (1.28–3.34)
	Q2	1.40 (0.88–2.23)	1.48 (0.90–2.42)
	Q3	1.10 (0.68–1.80)	1.16 (0.70–1.91)
	**Resolved comorbidity**		
	Q0 (lowest)	1.53 (1.15–2.03)	1.30 (0.92–1.85)

	Q1	1.51 (1.13–2.00)	1.34 (0.97–1.86)
	Q2	1.45 (1.10–1.93)	1.36 (1.00–1.84)
	Q3	1.25 (0.93–1.67)	1.19 (0.87–1.61)
**Parental Education (NVQ Levels)**	NVQ5 (Degree Level)	Reference	
	**No comorbidity**	Base outcome	
	**Persistent comorbidity**		
	Overseas/Others	1.50 (0.76–2.96)	1.23 (0.57–2.66)
	NVQ1	2.14 (1.00–4.61)	1.74 (0.76–3.97)
	NVQ2	2.25 (1.30–3.88)	1.80 (0.98–3.30)
	NVQ3	1.88 (1.06–3.32)	1.59 (0.85–2.97)
	NVQ4	1.46 (0.86–2.49)	1.33 (0.77–2.30)
	**Newly developed comorbidity**		
	Overseas/Others	1.60 (0.91–2.79)	1.02 (0.54–1.94)
	NVQ1	2.41 (1.31–4.43)	1.55 (0.79–3.04)
	NVQ2	1.60 (0.98–2.59)	1.11 (0.64–1.90)
	NVQ3	1.16 (0.68–1.98)	0.92 (0.52–1.63)
	NVQ4	1.16 (0.73–1.83)	1.07 (0.67–1.71)
	**Resolved comorbidity**		
	Overseas/Others	1.54 (1.04–2.28)	1.38 (0.87–2.18)
	NVQ1	1.41 (0.84–2.37)	1.21 (0.69–2.11)
	NVQ2	1.60 (1.15–2.23)	1.37 (0.95–1.98)
	NVQ3	1.46 (1.03–2.06)	1.29 (0.88–1.88)
	NVQ4	1.21 (0.88–1.66)	1.15 (0.83–1.59)
**Cumulative ACE score**	**ACE score 0**	Reference	
	**No comorbidity**	Base outcome	
	**Persistent comorbidity**		
	1	1.12 (0.77–1.63)	1.20 (0.81–1.78)
	2	1.50 (0.98–2.30)	1.67 (1.04–2.67)
	≥3	1.59 (0.98–2.58)	1.87 (1.06–3.29)
	**Newly developed comorbidity**		
	1	1.27 (0.89–1.83)	1.28 (0.89–1.84)
	2	1.51 (0.99–2.30)	1.44 (0.93–2.24)
	≥3	1.78 (1.18–2.69)	1.65 (1.02–2.67)
	**Resolved comorbidity**		
	1	1.16 (0.91–1.48)	1.20 (0.94–1.54)
	2	1.36 (1.03–1.80)	1.44 (1.07–1.93)
	≥3	1.34 (0.96–1.87)	1.46 (0.98–2.17)

Notes:

1.Estimates are based on multiple imputation using MICE (N = 10,353) and are survey-weighted.

2.Adjusted models include sex at birth, sexual identity, ethnicity, household income quintile, parental education (where applicable), and cumulative ACE score, with ‘no comorbidity’ as the reference category.

3.RRR = Relative Risk Ratio; CI = Confidence Interval; Std Error= Standard Error

4.NVQ levels: NVQ5 = university degree or higher; NVQ4 = higher education below degree; NVQ3 = A-level or equivalent; NVQ2 = GCSE level; NVQ1 = basic qualification; “Overseas/Other” = qualifications not mapped to the UK NVQ framework.

Supplementary Table 3. Multinomial Logistic Regression Predicting Comorbidity Change Status from Ages 14 to 17 Using Complete Case Analysis

Predictor	Category	SDQ-E Coef	SDQ-E (CI 95%)	Kessler-6 Coef	Kessler-6 (CI 95%)
**Sex at birth**	**No comorbidity**				
	Male	1	(1.00, 1.00)	1	(1.00, 1.00)
	Female	1	(1.00, 1.00)	1	(1.00, 1.00)
	**Persistent comorbidity**				
	Male	1	(1.00, 1.00)	1	(1.00, 1.00)
	Female	3.37	(2.41, 4.72)	3.41	(2.32, 5.00)
	**Newly developed comorbidity**				
	Male	1	(1.00, 1.00)	1	(1.00, 1.00)
	Female	2.04	(1.59, 2.62)	1.73	(1.28, 2.33)
	**Resolved comorbidity**				
	Male	1	(1.00, 1.00)	1	(1.00, 1.00)
	Female	1.57	(1.28, 1.93)	1.64	(1.35, 2.00)
**Ethnicity**	**No comorbidity**				
	White	1	(1.00, 1.00)	1	(1.00, 1.00)
	Mixed	1	(1.00, 1.00)	1	(1.00, 1.00)
	South Asian	1	(1.00, 1.00)	1	(1.00, 1.00)
	Black	1	(1.00, 1.00)	1	(1.00, 1.00)
	**Persistent comorbidity**				
	White	1	(1.00, 1.00)	1	(1.00, 1.00)
	Mixed	0.7	(0.24, 2.06)	0.64	(0.23, 1.74)
	South Asian	2.44	(0.80, 7.45)	1.64	(0.54, 4.95)
	Black	0.59	(0.11, 3.14)	0.49	(0.10, 2.52)
	**Newly developed comorbidity**				
	White	1	(1.00, 1.00)	1	(1.00, 1.00)
	Mixed	0.4	(0.20, 0.83)	0.46	(0.20, 1.02)
	South Asian	0.36	(0.13, 1.05)	0.5	(0.16, 1.50)
	Black	0.49	(0.17, 1.43)	0.38	(0.10, 1.44)
	**Resolved comorbidity**				
	White	1	(1.00, 1.00)	1	(1.00, 1.00)
	Mixed	0.53	(0.29, 0.98)	0.57	(0.31, 1.05)
	South Asian	0.91	(0.44, 1.87)	1.13	(0.55, 2.29)
	Black	0.4	(0.14, 1.11)	0.43	(0.15, 1.20)
**Sexual identity**	**No comorbidity**				
	Heterosexual	1	(1.00, 1.00)	1	(1.00, 1.00)
	Mainly heterosexual	1	(1.00, 1.00)	1	(1.00, 1.00)
	Bisexual	1	(1.00, 1.00)	1	(1.00, 1.00)
	Gay/Lesbian	1	(1.00, 1.00)	1	(1.00, 1.00)
	**Persistent comorbidity**				
	Heterosexual	1	(1.00, 1.00)	1	(1.00, 1.00)
	Mainly heterosexual	2.85	(1.96, 4.13)	2.39	(1.54, 3.71)
	Bisexual	4.1	(2.81, 6.00)	4.66	(3.09, 7.01)
	Gay/Lesbian	5.37	(3.11, 9.28)	5.12	(2.77, 9.48)
	**Newly developed comorbidity**				

	Heterosexual	1	(1.00, 1.00)	1	(1.00, 1.00)
	Mainly heterosexual	2.23	(1.62, 3.05)	2.97	(2.06, 4.29)
	Bisexual	2.64	(1.86, 3.73)	3.66	(2.47, 5.43)
	Gay/Lesbian	1.96	(1.04, 3.70)	1.79	(0.77, 4.15)
	**Resolved comorbidity**				
	Heterosexual	1	(1.00, 1.00)	1	(1.00, 1.00)
	Mainly heterosexual	1.54	(1.15, 2.06)	1.78	(1.36, 2.32)
	Bisexual	1.3	(0.89, 1.91)	1.36	(0.95, 1.94)
	Gay/Lesbian	1.75	(1.01, 3.02)	2	(1.22, 3.26)
**Income in quintiles**	**No comorbidity**				
	Q0 (lowest)	1	(1.00, 1.00)	1	(1.00, 1.00)
	Q1	1	(1.00, 1.00)	1	(1.00, 1.00)
	Q2	1	(1.00, 1.00)	1	(1.00, 1.00)
	Q3	1	(1.00, 1.00)	1	(1.00, 1.00)
	Q4	1	(1.00, 1.00)	1	(1.00, 1.00)
	**Persistent comorbidity**				
	Q0 (lowest)	1.64	(0.93, 2.90)	1.98	(1.03, 3.82)
	Q1	1.93	(1.16, 3.23)	1.93	(1.04, 3.57)
	Q2	1.58	(0.96, 2.61)	2.16	(1.21, 3.84)
	Q3	1.37	(0.84, 2.24)	1.71	(0.96, 3.05)
	Q4	1	(1.00, 1.00)	1	(1.00, 1.00)
	**Newly developed comorbidity**				
	Q0 (lowest)	1.94	(1.23, 3.05)	2.5	(1.45, 4.31)
	Q1	1.97	(1.30, 2.99)	2.14	(1.27, 3.58)
	Q2	1.62	(1.08, 2.42)	1.37	(0.81, 2.33)
	Q3	1.07	(0.71, 1.62)	1.03	(0.60, 1.76)
	Q4	1	(1.00, 1.00)	1	(1.00, 1.00)
	**Resolved comorbidity**				
	Q0 (lowest)	1.54	(1.04, 2.30)	1.46	(1.00, 2.14)
	Q1	1.4	(0.96, 2.02)	1.45	(1.03, 2.05)
	Q2	1.58	(1.13, 2.23)	1.42	(1.03, 1.97)
	Q3	1.18	(0.84, 1.66)	1.12	(0.81, 1.55)
	Q4	1	(1.00, 1.00)	1	(1.00, 1.00)
**Parental Education (NVQ Levels)**	**No comorbidity**				
	Overseas/Others	1	(1.00, 1.00)	1	(1.00, 1.00)
	NVQ1	1	(1.00, 1.00)	1	(1.00, 1.00)
	NVQ2	1	(1.00, 1.00)	1	(1.00, 1.00)
	NVQ3	1	(1.00, 1.00)	1	(1.00, 1.00)
	NVQ4	1	(1.00, 1.00)	1	(1.00, 1.00)
	NVQ5	1	(1.00, 1.00)	1	(1.00, 1.00)
	**Persistent comorbidity**				
	Overseas/Others	1.27	(0.60, 2.71)	1.54	(0.65, 3.63)
	NVQ1	1.51	(0.65, 3.49)	1.6	(0.59, 4.33)
	NVQ2	1.38	(0.76, 2.50)	1.85	(0.93, 3.70)
	NVQ3	1.85	(1.03, 3.32)	1.79	(0.88, 3.63)
	NVQ4	1.18	(0.70, 1.99)	1.41	(0.76, 2.64)
	NVQ5	1	(1.00, 1.00)	1	(1.00, 1.00)
	**Newly developed comorbidity**				

	Overseas/Others	1.37	(0.78, 2.42)	1.19	(0.61, 2.32)
	NVQ1	0.87	(0.42, 1.79)	1.24	(0.58, 2.66)
	NVQ2	0.94	(0.58, 1.53)	0.91	(0.51, 1.63)
	NVQ3	0.8	(0.48, 1.34)	0.85	(0.46, 1.56)
	NVQ4	1.15	(0.77, 1.73)	0.93	(0.57, 1.54)
	NVQ5	1	(1.00, 1.00)	1	(1.00, 1.00)
	**Resolved comorbidity**				
	Overseas/Others	1.5	(0.89, 2.51)	1.39	(0.85, 2.28)
	NVQ1	1.16	(0.61, 2.19)	1.21	(0.67, 2.19)
	NVQ2	1.4	(0.92, 2.14)	1.28	(0.86, 1.91)
	NVQ3	1.24	(0.80, 1.90)	1.34	(0.90, 2.00)
	NVQ4	1.21	(0.84, 1.74)	1.12	(0.80, 1.58)
	NVQ5	1	(1.00, 1.00)	1	(1.00, 1.00)
**Cumulative ACE score**	**No comorbidity**				
	0	1	(1.00, 1.00)	1	(1.00, 1.00)
	1	1	(1.00, 1.00)	1	(1.00, 1.00)
	2	1	(1.00, 1.00)	1	(1.00, 1.00)
	≥3	1	(1.00, 1.00)	1	(1.00, 1.00)
	**Persistent comorbidity**				
	0	1	(1.00, 1.00)	1	(1.00, 1.00)
	1	1.18	(0.67, 2.08)	1.14	(0.60, 2.14)
	2	1.38	(0.60, 3.19)	1.23	(0.46, 3.25)
	≥3	1.26	(0.85, 1.86)	1.14	(0.74, 1.77)
	**Newly developed comorbidity**				
	0	1	(1.00, 1.00)	1	(1.00, 1.00)
	1	1.53	(0.97, 2.42)	1.66	(0.91, 3.03)
	2	0.9	(0.38, 2.14)	1.6	(0.65, 3.98)
	≥3	1.44	(1.03, 2.02)	1.58	(1.01, 2.46)
	**Resolved comorbidity**				
	0	1	(1.00, 1.00)	1	(1.00, 1.00)
	1	1.05	(0.71, 1.56)	1.07	(0.73, 1.55)
	2	1.66	(0.99, 2.81)	1.72	(1.04, 2.84)
	≥3	1.01	(0.77, 1.33)	1.07	(0.82, 1.38)
